# Serial order learning of subliminal visual stimuli: evidence of multistage learning

**DOI:** 10.3389/fpsyg.2015.00076

**Published:** 2015-02-16

**Authors:** Kaede Kido, Shogo Makioka

**Affiliations:** ^1^Information Processing Center, Osaka Kyoiku UniversityKashihara, Japan; ^2^Department of Human Sciences, Osaka Prefecture UniversitySakai, Japan

**Keywords:** serial order learning, statistical learning, subliminal processing, binocular rivalry, continuous flash suppression (CFS)

## Abstract

It is widely known that statistical learning of visual symbol sequences occurs implicitly (Kim et al., 2009). In this study, we examined whether people can learn the serial order of visual symbols when they cannot detect them. During the familiarization phase, triplets or quadruplets of novel symbols were presented to one eye under continuous flash suppression (CFS). Perception of the symbols was completely suppressed by the flash patterns presented to the other eye [binocular rivalry (BR)]. During the test phase, the detection latency was faster for symbols located later in the triplets or quadruplets. These results indicate that serial order learning occurs even when the participants cannot detect the stimuli. We also found that detection became slower for the last item of the triplets or quadruplets. This phenomenon occurred only when the participants were familiarized with the symbols under CFS, suggesting that the subsequent symbols interfered with the processing of the target symbol when conscious perception was suppressed. We further examined the nature of the interference and found that it occurred only when the subsequent symbol was not fixed. This result suggests that serial order learning under BR is restricted to fixed order sequences. Statistical learning of the symbols’ transition probability might not occur when the participants cannot detect the symbols. We confirmed this hypothesis by conducting another experiment wherein the transition probability of the symbol sequence was manipulated.

## INTRODUCTION

The ability to learn and predict the sequence of events in the environment is important for organisms; it is also needed to perform higher order activities, such as language use. Sequence learning can be processed without awareness. For example, research has shown that adults can automatically learn complex artificial language without noticing the rules governing it (Reber, 1967, 1989). Similarly, children can rapidly learn the statistical properties of the outside world. Saffran et al. (1996) found that when infants as young as 8 months were exposed to a synthesized speech stream containing three-syllable words in random order, during a subsequent test phase they were more interested in novel words than in old words. These findings indicate that infants can learn the statistical properties of syllable sequences. Statistical speech learning has been reproduced in adults and children as well as infants (Saffran et al., 1997, 1999). This phenomenon is considered to be a domain-general and automatic mechanism which do not require intention or awareness (e.g., Baker et al., 2004; Toro et al., 2005).

The results of Saffran et al. (1996) have been confirmed in the visual domain. Fisher and Aslin (2001) showed that unsupervised visual statistical learning occurs on visual symbols’ spatial structure. In this study, the participants saw complex visual scenes that comprised pairs of unfamiliar symbols. Three pairs of symbols were presented randomly in one of nine spatial locations, with each pair always presented in the same spatial relationship. The participants did not focus attention on the symbols during the learning phase. However, they could distinguish novel and learned pairs at better than chance level. This result suggests that the participants implicitly learned the spatial relationship between symbols. Moreover, Kim et al. (2009) found that the serial order learning of visual stimuli’s temporal structure occurs without awareness. In their experiment, visual stimuli were presented consecutively (e.g., ABC-GHI-DEF-ABC-DEF…). The stimuli comprised triplets of novel symbols. The symbols’ order within each triplet was fixed, but in the stream, triplets were presented randomly. Results showed that the participants could learn the visual stimuli’s serial order. Although the participants were unaware of the stimuli order because they were presented rapidly, they were aware of the stimuli’s existence. Thus, whether serial order learning occurs when participants cannot detect visual stimuli remains unclear.

Turk-Browne et al. (2005) investigated the effect of attention on serial order learning. Red and green graphical symbols were presented in an interleaved sequence, and the participants were instructed to attend to only one color. After the learning phase, the familiarity of the attended stimuli increased, but that of the unattended stimuli remained unchanged. This result suggests that the learning of the unattended stimuli’s sequence does not occur. While this lack of learning might be caused by the absence of perception, there remains the possibility that the processing for unattended stimuli is disturbed by that of the attended stimuli (Raymond et al., 1992). Furthermore, as Kim et al. (2009) pointed out, explicit learning of stimuli might affect the result of the familiarity test used by Turk-Browne et al. (2005).

Herein, to completely exclude the effect of explicit learning, we examined the nature of serial order learning when the participants could not detect the stimuli at all. To prevent the perception of the stimuli for several seconds, we used the continuous flash suppression (CFS) paradigm (Tsuchiya and Koch, 2005; Tsuchiya et al., 2006). When different visual stimuli are presented to each eye, one stimulus is suppressed from awareness, and the other is detected dominantly; this phenomenon is called binocular rivalry (BR). The CFS paradigm is known to produce stable BR. Flash streams presented to one eye suppress, for more than 10 s, the perception of the stimuli presented to the other eye. We investigated whether serial order learning occurred for the symbol sequence presented to the suppressed eye.

Although the information from the suppressed eye does not reach consciousness, there is evidence for the occurrence of a relatively higher order processing during CFS. Firstly, the representation of familiar stimuli, such as letters or faces, was activated (Jiang et al., 2007; Costello et al., 2009). Secondly, repetition and cross-script priming for words were observed (Kido and Makioka, 2014). Thirdly, processing for tools was found to occur in the dorsal visual pathway (Fang and He, 2005; Almeida et al., 2008). However, whether people can learn the sequence of stimuli under BR remains unknown.

In Experiment 1, we examined whether serial order learning of novel symbol sequences occurred during BR. We also investigated whether the learning depended on the eye exposed to the stimuli. If the learning depends on the eye, then the learning happens at the monocular level of visual processing. The learning of the last symbol in each triplet was found to be impeded under BR which is a novel phenomenon. Thereafter, we examined its possible cause in Experiments 2 and 3. In Experiment 2, we investigated whether limitation of memory span caused the interference. In Experiments 3 and 4, we examined the effect of symbol sequences’ transition probability on learning under BR.

## EXPERIMENT 1

We investigated whether serial order learning occurred when the participants could not be aware of the stimuli. Herein, streams of novel symbols were presented on a screen during the familiarization phase. The participants could not detect the symbols due to the CFS. If they could learn the serial order of the symbols presented subliminally, their responses in the subsequent target detection task would become faster because target symbols were presented in the same triplet as in the familiarization phase.

To explore the mechanism of subliminal serial order learning, we also examined the effect of eye congruency between the familiarization and test phases. If the effect of serial order learning is greater when the stimuli are presented to the same eye in both the familiarization and the test phases, the learning happens at the monocular level (i.e., the very early level of visual processing).

### METHODS

#### Participants

Thirty-six graduate and undergraduate students (14 males, aged 18–24, average 20.4 years) participated. All were native Japanese speakers and had normal or corrected-to-normal vision. The data of four participants (three males) were excluded from the analysis because these participants could not converge the visual field of their eyes. All participants gave informed consent in accordance with the Ethical Review Board of Osaka Prefecture University.

#### Stimuli

**FIGURE 1:**
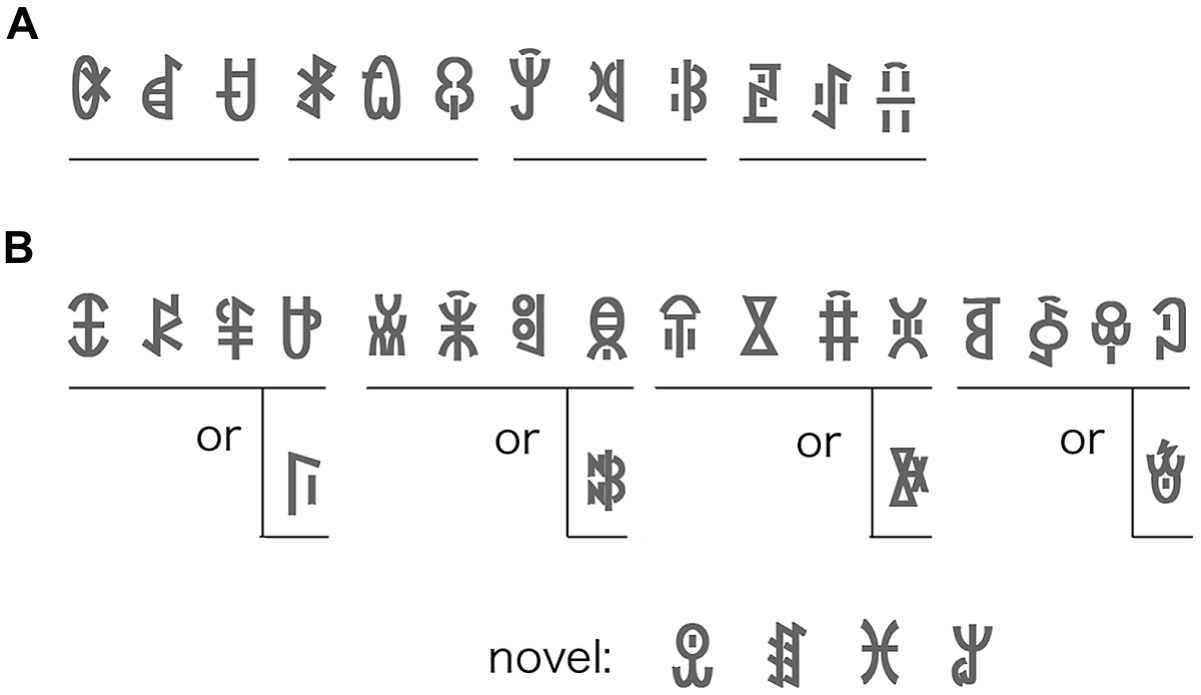
**(A)** An example of the symbol sequence in Experiment 1. Four triplets (12 symbols) were used both in the familiarization and test phases. The symbol order within each triplet was fixed, and the triplets were presented in random order. **(B)** An example of symbol sequence in Experiment 2. Four quadruplets (16 symbols) were used in the familiarization phase. Four novel symbols were added in the test phase. The symbol order within each quadruplet was fixed (symbols only in the upper row) in Experiment 2, and the quadruplets were presented in random order. In the 50% condition of Experiment 3, the fourth quadruplet symbol was selected randomly from the upper or lower row.

Twenty-four different symbols of modern Yi script were selected and grouped into eight triplets (**Figure 1**). All symbols were novel for all the participants. The symbols were presented within a bound of 5 ° × 5° of visual angle (60 × 60 pixels). The flash stream consisted of three types of shapes (oval, triangle, and rectangle). These shapes and their colors (red, blue, and green) were randomly selected in each flash pattern. A flash stream, within a bound of 15° × 15° of visual angle, was composed of 300 randomly generated patterns. The symbols were drawn in black against a gray background. To prevent the transition of the symbols breaking the suppression, the contrast between the symbols and background was set very low (8%). A Gaussian filter with a radius of 5 pixels blurred the symbols’ contours.

Stimuli were presented on a CRT display (NANAO Flex Scan 54T) at a 75 Hz refresh rate. These stimuli were generated and presented by MATLAB with Psychophysics Toolbox (Brainard, 1997; Pelli, 1997), running on a personal computer (Apple Mac mini, 1.66 GHz Intel Core Duo). The display was located 43 cm from the participants’ eyes. In the display, the luminance of the black and white areas was 0.81 cd/m^2^ and 105.94 cd/m^2^, respectively. The participants viewed the display through a mirror stereoscope mounted on a chin rest.

#### Procedure

**FIGURE 2:**
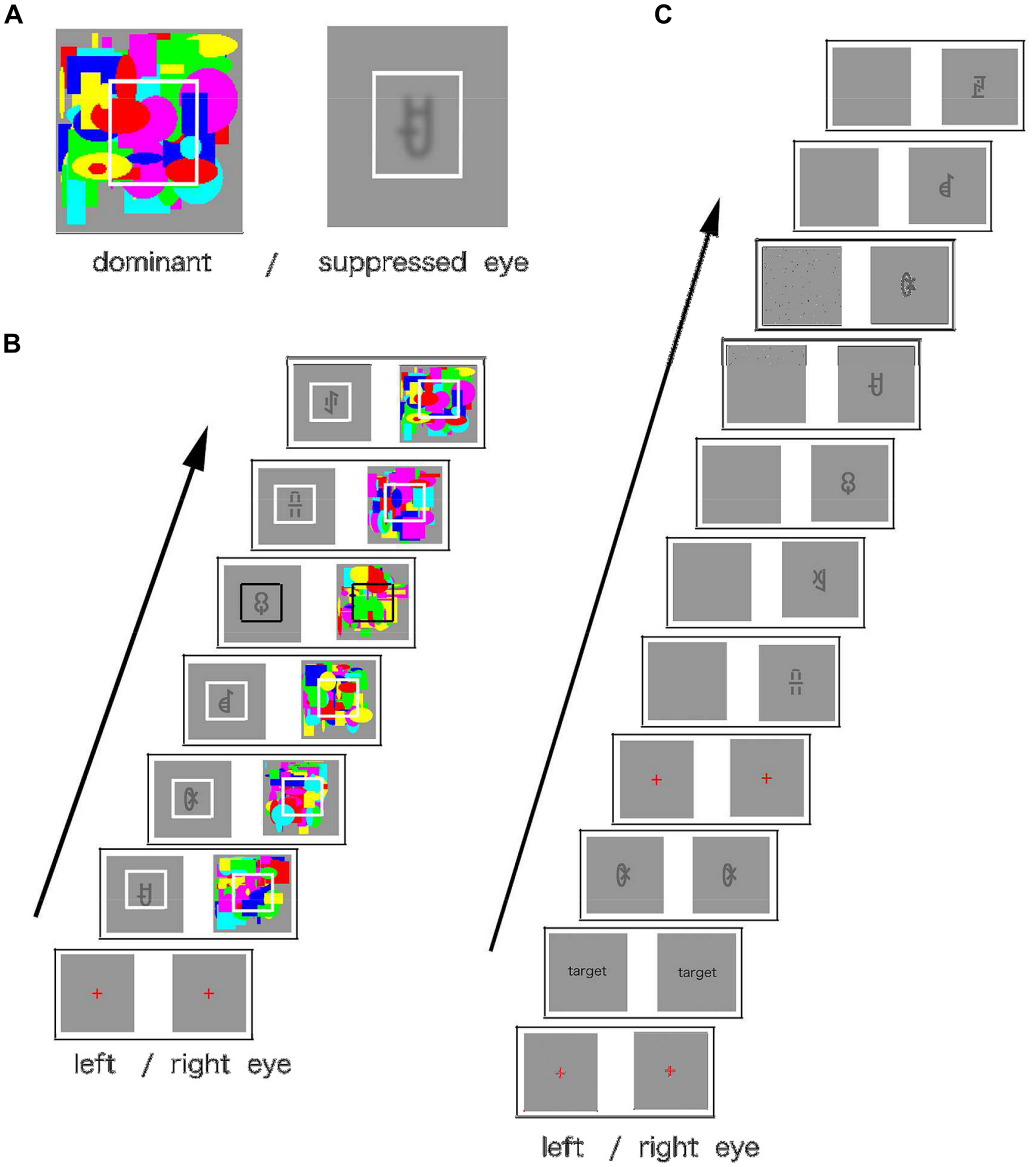
**(A)** An example of stimulus configuration. Participants saw the display through a stereoscope. A random flash pattern was presented to one eye (dominant eye), and a symbol (Yi script) was presented to the other eye (suppressed eye). White frames were presented to both eyes to aid the eyes’ convergence. **(B)** The sequence of stimuli in the familiarization phase. The symbols were updated every 400 ms, and the flash stream was updated every 200 ms. Participants were instructed to press the space key when the white frame turned black. **(C)** The sequence of the stimuli in the test phase. The target symbol was presented to both eyes, until the participant pressed the space key. The target then disappeared, and the test stream was presented to one eye. In the congruent eye condition, symbols were presented to the same eye as in the familiarization phase or, in the incongruent eye condition, to the other eye. The test stream was constructed as follows: it consisted of 12 symbols presented in the familiarization phase. The triplet containing the target symbol was presented in the same order as in the familiarization phase, and the order of the other symbols was randomized. The triplet was not presented in the stream’s first or last position. In Experiments 2 and 3, quadruplets were used instead of triplets. The participants were told to press the space key as quickly as possible when they detected the target symbol.

Each experiment consisted of two phases: familiarization and test. During the familiarization phase, using one eye, the participants viewed a rapid serial visual presentation (RSVP) of a continuous stream of visual symbols. A symbol stream consisted of four types of triplets, each presented 100 times in a stream (total presentation time approximately 15 min). The triplets’ presentation order was randomized; however, the items’ order within triplets remained constant. The participants could not detect the symbol stream because the continuous flash stream was presented to the other eye (CFS). Each visual symbol was shown for 400 ms, and the inter-stimulus interval (ISI) was set to zero. A random flash pattern was shown for 200 ms with no ISI (see **Figure 2**). The participants were instructed to press the space key when the white frame, presented around a symbol, changed briefly to black. This task focused the participants’ attention on the center of the screen. All participants were unaware of the symbols in the familiarization phase. The eye presented with the symbols remained constant during the familiarization phase, and the presentation eye was counterbalanced among the participants.

The familiarization phase was followed by the RSVP target detection test phase. A target symbol was presented to one eye until the key press, and a sequence of 24 symbols (target detection sequence) was presented to the same eye. In the target detection sequence, each symbol was presented for 400 ms with no ISI. Since no stimuli were presented to the other eye, the participants could detect the symbols. They were asked to press the space key as soon as they detected the target symbol in the sequence. The target symbol was always contained in the same triplet as that presented in the familiarization phase. The order of the other symbols was randomized, and the triplets containing the target began at either the fourth or seventh position in the target detection sequence. The position of the triplets was assigned randomly and counterbalanced among the participants. Forty-eight test trials were conducted.

After the test phase, a perceptibility test was conducted to confirm that the participants could not detect the visual symbols. The same visual symbols as in the familiarization phase were shown for 400 ms to one eye, and the flash stream was presented to the other eye (subliminal exposure). Two-alternative forced choice (2AFC) was then conducted. The participants pressed a key (left or right arrow key) to indicate which of two symbols they felt had been presented in the subliminal exposure. Twenty-four test trials were conducted.

### RESULTS

**FIGURE 3:**
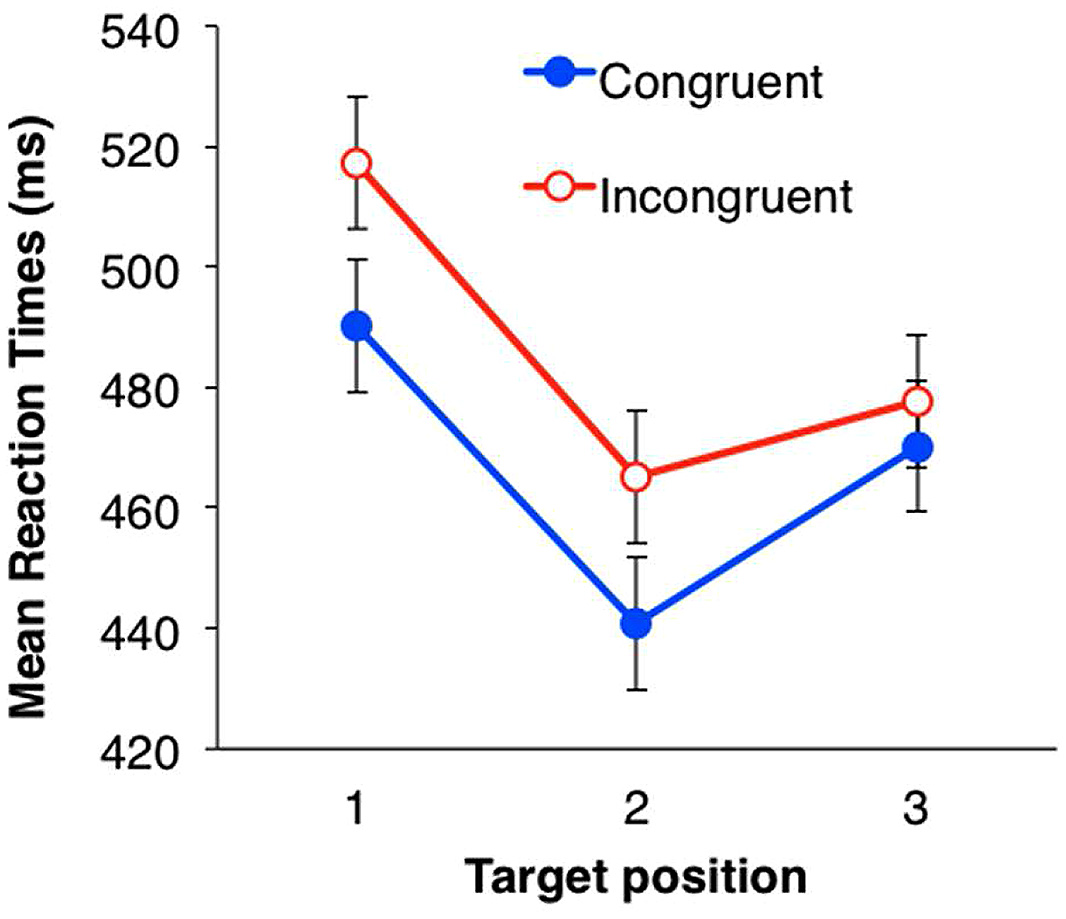
**Mean RTs of target detection in Experiment 1.** Filled circles (blue) indicate the mean RTs in the congruent eye condition; open circles (red) indicate the mean RTs in the incongruent condition. Error bars represent SE.

During the test phase, we analyzed the mean response times (RTs) for the target detection task as a dependent variable. Incorrect responses and responses with an RT below 200 ms (1.9%) were excluded from the analysis. Mean RTs in each condition are shown in **Figure 3**. The RT in each trial was converted by a logarithmic transformation, because the data distribution was positively skewed (2.33). Within-subjects ANOVAs were performed with a two (eye congruency: congruent vs. incongruent) × three (serial position in the triplets) design. Both the main effects were significant [eye congruency; *F*(1,31) = 20.32, *p* < 0.001, partial η^2^ = 0.40, serial position; *F*(2,62) = 50.83, *p* < 0.001, partial η^2^ = 0.62]. The mean RT was significantly shorter when the presentation eye was congruent between the familiarization and the test phases. The interaction between eye congruency and serial position was not significant [*F*(2,62) = 0.16, *n.s.*]. Multiple comparison was conducted for the main effect of serial position (Shaffer’s modified sequentially rejective Bonferroni procedure, *p* < 0.05) and revealed that the mean RTs for the second and third symbols in the triplets were significantly shorter than that for the first [1 > 2: *t*(31) = 9.71, Cohen’s *d* = 0.94; 1 > 3: *t*(31) = 6.14, Cohen’s *d* = 0.54], and the mean RT for the third symbol was significantly longer than that for the second symbol [2 < 3: *t*(31) = 4.13, *d* = 0.39].

The percentage rate of incorrect responses in all experiments is shown in **Table 1**. ANOVAs were performed on an angular transformed percentage of incorrect responses for each condition. Neither main effects nor interaction was significant [eye congruency: *F*(1,31) = 0.59, *n.s.*; serial position: *F*(2,62) = 0.69, *n.s.*; interaction: *F*(2,62) = 0.90, *n.s.*]. There was no evidence of speed–accuracy trade-off or effects of task difficulty on RT.

**Table 1:** Percentage of error rates in all experiments.

		Binocular rivalry (BR)				Non-binocular rivalry (NBR)			
Experiment and condition		Congruent		Incongruent		Congruent		Incongruent	
		*M*	SE	*M*	SE	*M*	SE	*M*	SE
Experiment 1	Position 1	1.95	0.99	1.17	0.65				
	Position 2	1.17	0.65	2.34	0.88				
	Position 3	1.95	0.82	2.73	0.93				
Experiment 2	Position 1	2.08	1.23	1.56	0.86	1.88	1.02	0.00	0.00
	Position 2	1.56	1.14	2.08	0.97	1.88	1.02	1.25	1.25
	Position 3	3.65	1.92	2.08	0.97	2.50	1.94	1.88	1.37
	Position 4	2.08	0.97	1.04	0.72	1.88	1.02	1.25	0.86
	Novel	1.04	0.72	1.88	1.02	3.13	1.24	1.88	1.37
Experiment 3	100%	2.50	1.38	1.67	1.15	1.00	1.00	3.00	2.19
	50%	1.67	1.15	4.17	2.08	2.00	1.38	5.00	1.99
	Blank	3.33	1.97	3.33	1.55	1.00	1.00	2.00	1.38
	Novel	0.83	0.83	2.50	1.38	6.00	2.55	2.00	1.38
Experiment 4	Rulel	3.13	1.39	1.56	0.87	3.33	1.36	2.00	1.11
	Rule2	1.56	0.87	2.08	0.99	3.33	1.36	2.67	1.25
	Rule3	1.56	0.87	2.60	1.09	2.67	1.25	2.67	1.25
	Novel	2.08	0.99	6.25	1.79	3.33	1.36	6.00	1.90

To analyze whether the participants were aware of the visual symbols in the familiarization phase, we counted the number of correct 2AFC judgments in the perceptibility test. The average percentage of correct responses for all participants was 54%; this rate is not significantly higher than the chance level[*t*(31) = 1.51, *n.s.*].

### DISCUSSION

The finding that target detection was faster for the second and third positions than for the first position suggests that detection of the latter symbols was facilitated, that is, serial order learning occurred under BR. As far as we know this is the first evidence of the serial order learning of subliminal visual symbols.

The response to the third symbol, however, was slower than to the second symbol. This result differed from those of Kim et al. (2009) and other experimenters on the statistical learning of the sequence of the stimuli (e.g., Saffran et al., 1996). Response latency usually decreases monotonically with the target’s serial position. One difference between Experiment 1 and Kim et al.’s (2009) experiment was the ISI between symbols in the familiarization phase. ISI was set to zero in our experiment, but there was a 30 ms interval between the symbols in Kim et al.’s (2009) experiment. In our experiment, the subsequent symbol might have interfered with the perception of the third symbol. When the target symbol was presented in the first or second triplet position, subsequent symbols were constant because the order of the symbols within the triplet was fixed. However, in the third position, subsequent symbols varied because the order between triplets was randomized. Therefore, variant subsequent symbols might interfere with the perceptual process for target symbols.

Target detection was faster when the stimuli were presented to the same eye in both the familiarization and the test phases. The suggestion is that the familiarization phase facilitates processing at the monocular level. However, interaction between serial order and eye congruency was not observed. Thus the implication is that eye congruency only affects the overall latency of target detection, which suggests that serial order learning does not depend on monocular level processing. In the congruent eye condition, the overall facilitation’s cause might lie in the adaptation process at the monocular level. In fact, V1 neurons change their orientation preference after short-term exposure to a stimulus (Dragoi et al., 2000). If the monocular neurons in V1 change their tuning to the symbols presented during the familiarization phase, target detection by the same eye should be facilitated.

## EXPERIMENT 2

Limitation of the learning mechanism’s memory span might cause a delayed target detection in the triplets’ third position. To verify whether the delay originated from a limited memory span or interference by subsequent symbols, we changed the stimuli from triplets to quadruplets. If a limited memory span caused the delay, the detection latency for the quadruplets’ third symbol should be slower than that for the second symbol. In contrast, if target detection were reduced by the interference, only the last symbol’s latency would be delayed.

To compare the subliminal and supraliminal processes, we added a condition wherein the perception was not suppressed by BR [Non-binocular rivalry (NBR) condition]. If the interference occurs only during the subliminal process, the last symbol’s delay should be observed only in the BR condition.

### METHODS

#### Participants

Forty-six graduate and undergraduate students participated (aged 19–36, average 22.5 years), 20 students in the NBR condition (six males) and 26 in the BR condition (six males). All were native Japanese speakers and had normal or corrected-to-normal vision. The data of two participants (BR condition) were excluded from the analysis, since they could not converge the visual fields of their eyes. None had participated in Experiment 1.

#### Stimuli and procedure

The stimuli and procedures were the same as those in Experiment 1, except for the following: (1) In the familiarization phase, stimuli consisted of quadruplets instead of triplets (**Figure 2B**). (2) The NBR condition was added. In this condition, RVSP of a continuous stream of visual symbol sequences was presented to one eye, and in the familiarization phase, no stimuli were shown to the other eye. The participants could detect the symbol stream but were unaware of the stimuli order because stimuli were presented rapidly. The test phase was the same as that for the BR condition, but the perceptibility test was not conducted. (3) In the test phase, to examine the effect of the target symbols’ familiarity, we added novel symbols as target stimuli. One quadruplet was selected randomly, and one symbol in the quadruplets was replaced with a novel symbol. The novel symbol was presented equally across all four serial positions. (4) The stimuli consisted of 36 different symbols (eight quadruplets and four novel symbols).

### RESULTS

**FIGURE 4:**
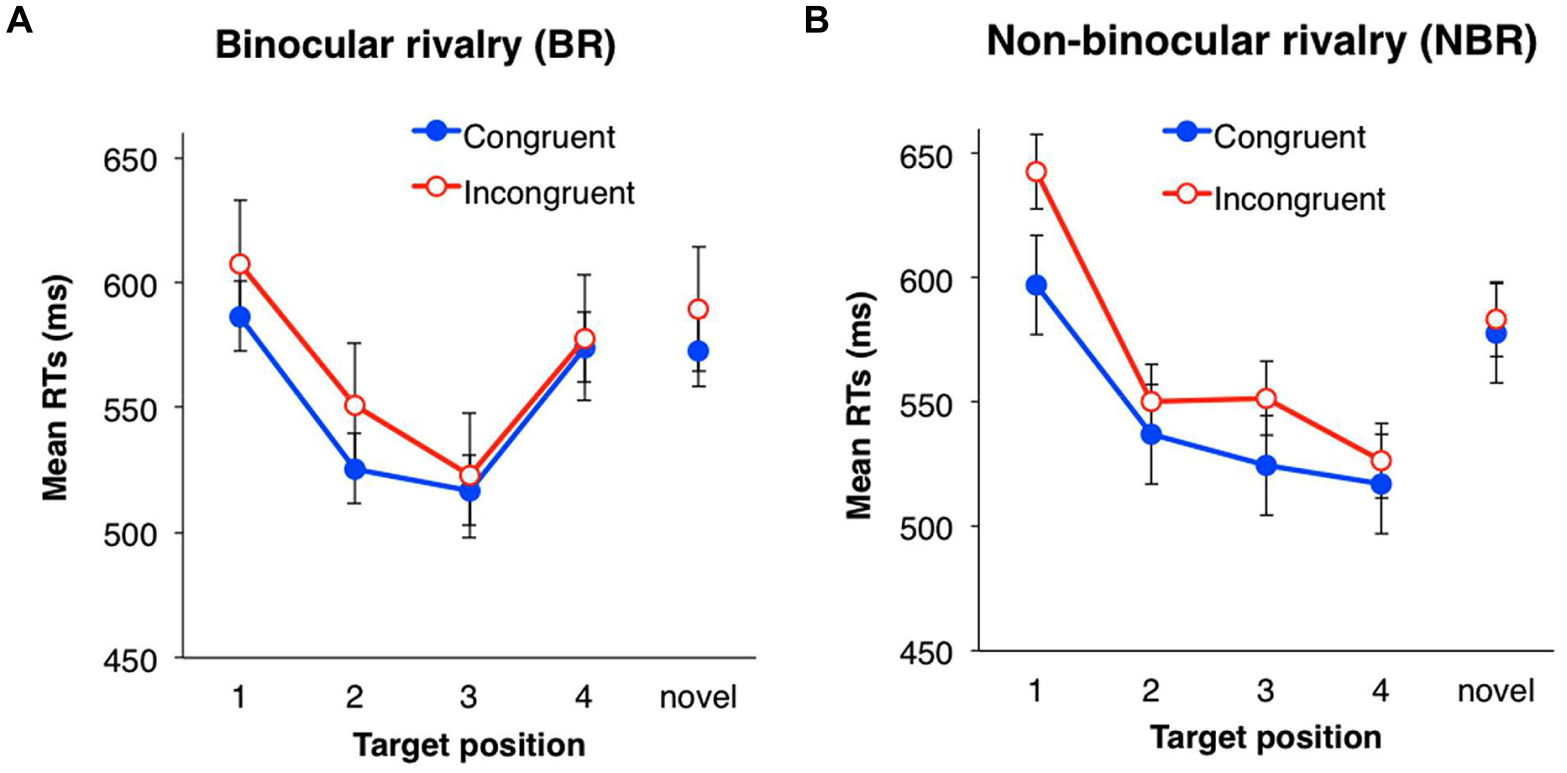
**Mean RTs of target detection in Experiment 2. (A)** Binocular rivalry (BR) condition. **(B)** Non-binocular rivalry (NBR) condition. Filled circles (blue) indicate the mean RTs in the congruent eye condition; open circles (red) indicate the mean RTs in the incongruent condition. Error bars represent SE.

Mixed ANOVAs (one between-subject and two within-subject design) were performed with a two (suppression: BR vs. NBR) × two (eye congruency; familiarized vs. unfamiliarized) × four (serial position: 1, 2, 3, and 4) design. Incorrect responses and responses with RT below 200 ms (2.1%) were excluded from analysis. In each trial, the RT was converted by a logarithmic transformation because the distribution of all data was positively skewed (5.38). Mean RTs for each condition are shown in **Figure 4**. The main effects of eye congruency and serial position were significant [eye congruency: *F*(1,42) = 4.60, *p* < 0.05, partial η^2^ = 0.10; serial position: *F*(3,126) = 13.96, *p* < 0.001, partial η^2^ = 0.25], but the main effect of suppression was not [*F*(1,42) = 0.46, *n.s.*]. The mean RT was significantly shorter when the presentation eye was congruent between the familiarization and testing phases.

The interaction between suppression and serial position was significant [*F*(3,126) = 3.90, *p* < 0.05, partial η^2^ = 0.09]. ANOVAs for simple main effects revealed that the main effects of serial position were significant in both BR and NBR conditions: [BR: *F*(3,69) = 7.88, *p* < 0.001, partial η^2^ = 0.26; NBR: *F*(3,57) = 10.11, *p* < 0.001, partial η^2^ = 0.35]. Multiple comparison (Shaffer’s modified sequentially rejective Bonferroni procedure, *p* < 0.05) was conducted for the main effect of serial position in each suppression condition. The BR condition showed a significant difference between positions one and two [1 > 2: *t*(23) = 2.74, *d* = 0.43], one and three [1 > 3: *t*(23) = 4.34, *d* = 0.63], and three and four [3 < 4: *t*(23) = 3.52, *d* = 0.52]. The NBR condition showed a significant difference between positions one and two [1 > 2: *t*(19) = 3.86, *d* = 0.45], one and three [1 > 3: *t*(19) = 4.32, *d* = 0.63], and one and four [1 > 4: *t*(19) = 4.06, *d* = 0.61]. In the BR condition, the RT of the last symbol (position four) was significantly longer than that of the third position, but in the NBR condition, there was no significant difference between the third and fourth positions. No other interactions were significant [suppression × eye congruency: *F*(1,42) = 1.92, *n.s.*; eye congruency × serial position: *F*(3,126) = 0.58, *n.s.*; three-way: *F*(3,126) = 0.78, *n.s.*].

To analyze whether symbol familiarity affected target detection, we compared the RT of the novel stimuli with that of the familiarized stimuli in the first serial position. Mixed ANOVAs were performed with a two (suppression: BR vs. NBR) × two (eye congruency: congruent vs. incongruent) × two [symbol familiarity: familiarized (position 1) vs. novel stimuli] design. None of the main effects were significant [suppression: *F*(1,42) = 0.06; *n.s.*; eye congruency: *F*(1,42) = 0.72, *n.s.*; symbol familiarity: *F*(1,42) = 0.02, *n.s.*]. No interactions were significant [suppression × eye congruency: *F*(1,42) = 0.71, *n.s.*; suppression × symbol familiarity: *F*(1,42) = 0.74, *n.s.*; three-way:*F*(1,42) = 0.08, *n.s.*].

ANOVAs were performed on the angular transformed percentage of incorrect responses for each condition. Neither main effects nor interaction was significant [suppression: *F*(1,42) = 0.90, *n.s.*; eye congruency: *F*(1,42) = 0.82, *n.s.*; serial position: *F*(3,126) = 0.22, *n.s.*; suppression × eye congruency: *F*(1,42) = 1.52, *n.s.*; suppression × serial position: *F*(3,126) = 0.46, *n.s.*; eye congruency × serial position: *F*(3,126) = 0.05, *n.s.*; three-way: *F*(3,126) = 0.94, *n.s.*]. There was no evidence of speed–accuracy trade-off or effects of task difficulty on RT.

To analyze whether the participants could detect visual symbols in the familiarization phase, we counted the number of correct responses in the perceptibility test; the average percentage of correct responses in the BR condition was 54%. This rate was not significantly higher than that of the chance level [*t*(23) = 0.73, *n.s.*], implying that the participants could not detect the symbols in the BR condition.

### DISCUSSION

The effect of serial order was observed in both BR and NBR conditions. In the BR condition, the response to the last symbol was slower than that to the third symbol. Experiment 1 showed the same tendency, where triplets of symbols were used. This result suggests that the response delay is not due to the memory limitation of the learning mechanism, rather due to the interference from subsequent symbols. In contrast, the response to the third symbol was faster than that to the first symbol, suggesting that serial order learning occurred for the third symbol.

In the NBR condition, the response to the last symbol was not delayed. The suggestion is that the interference only occurred when the symbols were presented subliminally. As the test-phase procedure was the same in both BR and NBR conditions, this indicates that the interference occurred in the familiarization phase. In the BR condition, target detection was facilitated if the symbol transition was fixed in the familiarization phase (the second and third symbols in the quadruplet) and target detection became slower when the symbol transition was not fixed in the familiarization phase (the last symbol in the quadruplet). These results suggest that the learning under BR occurs only when the sequence is fixed.

However, the RT for the first symbol in the familiarized sequence was not faster than that of the novel symbol. This result appears to contradict the result that target detection in the congruent condition was faster than that in the incongruent condition. If familiarization at the monocular level facilitates target detection by the same eye, the same process would also facilitate target detection of the familiarized sequence’s first symbol. This contradiction is resolved when it is postulated that familiarization at the monocular level occurs for overall visual properties, such as spatial frequency. In the eye-congruent condition, the symbols were presented to the same eye in both familiarization and test phases. In the incongruent condition, the participants saw target symbols with the eye exposed to the flash stream in the familiarization phase. If monocular level processing were modulated to the overall visual properties of the symbols or flash patterns, rather than to the specific property of each symbol, the overall effect of eye congruency should be observed. However, the detection of the first symbol should not be facilitated. The results of Experiment 2 support these views.

## EXPERIMENT 3

The result of Experiment 2 confirmed that detection delay of the last symbol was not caused by a limitation of memory span. Since the triplets (or quadruplets) were presented in random order, the subsequent symbol was not fixed at the last location. The suggestion is that the interference by the subsequent symbol in the familiarization phase became greater when the symbol sequence was not fixed. To verify the hypothesis that the interference was caused by the varying symbols following the target, we examined the effect of the symbol sequences’ transition probability in the familiarization phase.

In this experiment, the third position was fixed for the target symbols in the quadruplets. In the 100% (fixed) condition, the symbols and their order were fixed throughout the experiment. In the 50% (varied) condition, there were two candidates for the fourth symbol, and one candidate was selected randomly. In the blank condition, no symbol was presented after the target symbol. If the interference under BR decreases when the sequence is fixed, the response to the target in the 100% condition should be faster than that in the 50% condition. If the interference vanishes completely in the fixed sequence, the latency in the blank condition should be the same as that in the 100% condition. The response speed in those three conditions should be the same under NBR, because interference by the subsequent symbols did not occur in the NBR condition of Experiment 2.

### METHODS

#### Participants

Forty-four graduate and undergraduate students participated (aged 19–24, average 20.8 years); 20 students participated in the NBR group (12 males), and the other 24 participated in the BR group (8 males). All were native Japanese speakers and had normal or corrected-to-normal vision. None had participated in Experiments 1 or 2.

#### Stimuli and procedure

The stimuli and procedures were the same as those in Experiment 2 with the following exceptions. In the 100% condition, the last symbol in the quadruplets was always the same; in the 50% condition, one of the two symbols was randomly selected as the last symbol; in the blank condition, no symbol was presented in the fourth serial position. Twelve types of quadruplets of Yi script were selected for the familiarization phase. The stimuli consisted of 52 different symbols (16 symbols for the 100% condition, 20 for the 50% condition, 12 for the blank condition, and 4 novel symbols). Finally, target symbols appeared only in the third position in both familiarization and test phases. In the test phase, the novel symbol’s position was also fixed at the third position.

### RESULTS

**FIGURE 5:**
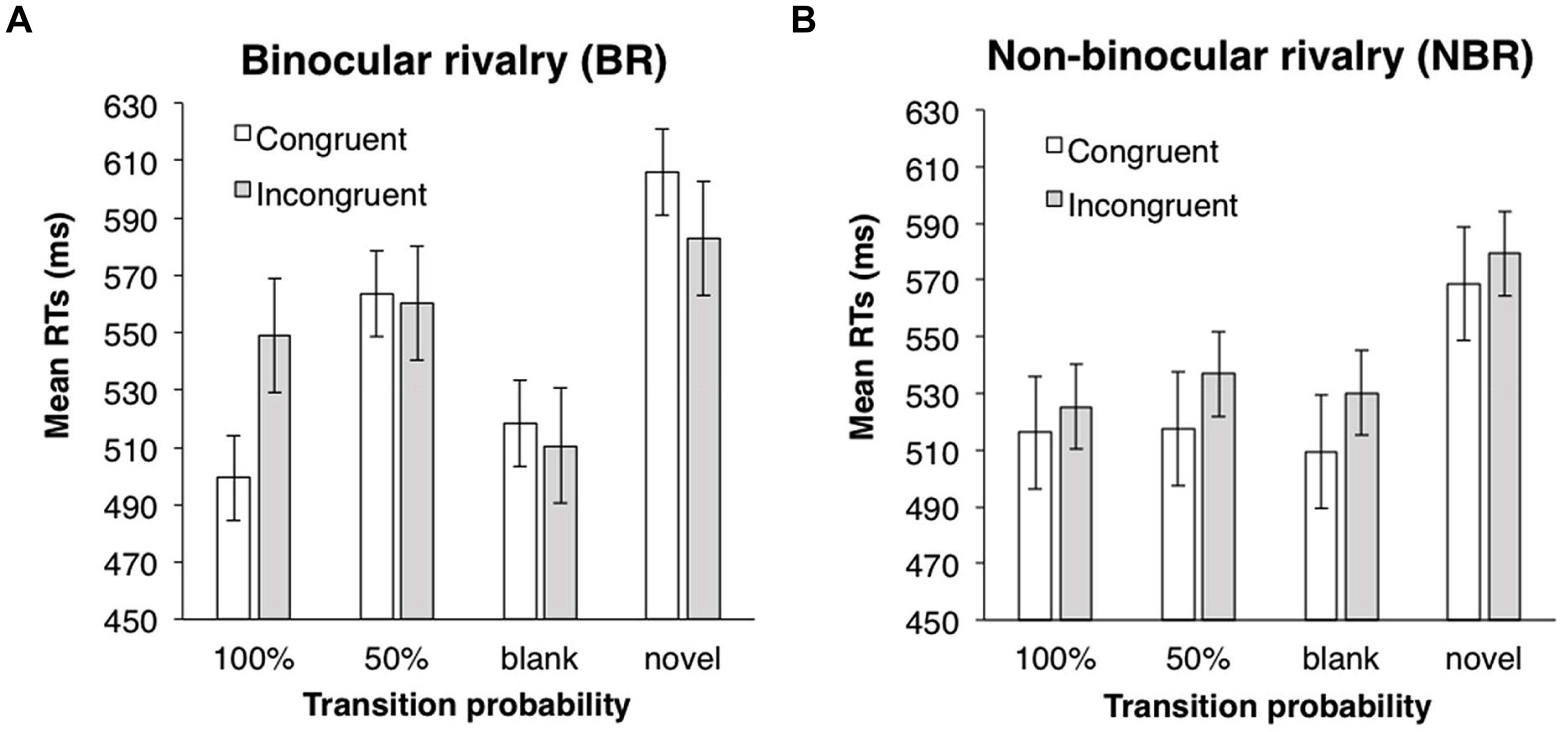
**Mean RTs of target detection in Experiment 3. (A)** BR condition. **(B)** NBR condition. White bars indicate the mean RTs in the congruent eye condition; gray bars indicate the mean RTs in the incongruent eye condition. Error bars represent SE.

ANOVAs (one between-subject and two within-subject design) were performed with a 2 (suppression: BR vs. NBR) × 2 (eye congruency: congruent vs. incongruent) × 3 (transition probability: 0.5, 1.0, and blank) design. Incorrect responses and responses with RT below 200 ms (2.7%) were excluded from the analysis. In each trial, the RT was converted by a logarithmic transformation, because the distribution of all data was positively skewed (4.20). Mean RTs for each condition are shown in **Figure 5**. The main effects of eye congruency and transition probability were significant (eye congruency; *F*(1,42) = 5.21, *p* < 0.05, partial η^2^ = 0.11; transition probability; *F*(2,84) = 7.49, *p* < 0.01, partial η^2^ = 0.15), but the main effect of suppression was not [*F*(1,42) = 0.13, *n.s.*]. The interaction between suppression and transition probability was significant [*F*(2,84) = 4.02, *p* < 0.05, partial η^2^ = 0.09]. ANOVAs for simple main effects revealed that the main effect of transition probability was significant in the BR condition, but not in the NBR condition [BR condition: *F*(2,46) = 10.13, *p* < 0.001, partial η^2^ = 0.31; NBR condition: *F*(2,38) = 0.41, *n.s.*]. Multiple comparison (Shaffer’s modified sequentially rejective Bonferroni procedure, *p* < 0.05) was conducted on the main effect of transition probability in the BR condition. There was a significant difference between the 50 and 100% conditions [50% > 100%: *t*(23) = 3.07, *d* = 0.60] and between the 50% and blank conditions [50% > blank: *t*(23) = 4.34, *d* = 0.75]. In the 50% condition, the mean RT was significantly longer than that in other transition probability conditions. However, in the NBR condition, transition probability had no significant effect. RTs were significantly shorter when the presentation eye was congruent between the familiarization and test phases. None of the other interactions were significant [suppression × eye congruency: *F*(1,42) = 0.72, *n.s.*; eye congruency × transition probability: *F*(2,84) = 0.57, *n.s.*; three-way: *F*(2,84) = 1.71, *n.s.*].

To confirm the learning effect, RTs in the 50%, 100%, and blank conditions were compared with RTs to the novel stimuli via paired two-tailed *t*-tests (eye congruency conditions were collapsed). In the BR condition, RTs in the 100% and blank conditions were significantly shorter than RTs to the novel symbols; RTs in the 50% condition did not differ from those in the novel symbols [100% vs. novel: *t*(46) = 2.56, *p* < 0.05, *d* = 0.74; 50% vs. novel: *t*(46) = 0.94, *n.s.*; blank vs. novel: *t*(46) = 2.98, *p* < 0.05, *d* = 0.70]. In the NBR condition, RTs in all the three conditions were significantly shorter than those in the novel symbols [100% vs. novel: *t*(38) = 2.88, *p* < 0.05, *d* = 0.91; 50% vs. novel: *t*(38) = 2.32, *p* < 0.05, *d* = 0.74; blank vs. novel; *t*(38) = 2.85, *p* < 0.05, *d* = 0.90]. A learning effect was observed except in the 50% condition under BR.

ANOVAs were performed on the angular transformed percentage of incorrect responses for each condition. Neither main effects nor interaction was significant [suppression; *F*(1,42) = 0.82, *n.s.*, eye congruency; *F*(1,42) = 1.26, *n.s.*, transition probability; *F*(2,84) = 0.52, *n.s.*; suppression × eye congruency: *F*(1,42) = 0.26, *n.s.*; suppression × transition probability: *F*(2,84) = 0.42, *n.s.*; eye congruency × transition probability: *F*(2,84) = 0.90, *n.s.*; three-way: *F*(2,84) = 0.25, *n.s.*]. There was no evidence of speed–accuracy trade-off or effects of task difficulty on RT.

We counted the number of correct responses in the perceptibility test. The average percentage of correct responses in the BR condition was 52%, not significantly higher than that of the chance level [*t*(23) = 0.62, *n.s.*].

### DISCUSSION

In the BR condition, target detection in the 50% condition was slower than in the 100% condition, and latency in the 100% condition did not differ from that in the blank condition. These results confirmed the hypothesis that varying symbols following the target caused an interference. Learning of the fixed symbol sequence not only facilitated detection of symbols (location 2 in Experiment 2 and locations 2 and 3 in Experiment 3) but also eliminated the subsequent symbol’s interference. However, transition probability had no effect in the NBR condition. The suggestion is that the interference occurs only when symbols are presented subliminally. We will further consider the learning mechanism under BR in General Discussion.

Mean RTs for the novel symbols were compared with those for targets. Note that in Experiment 3, targets were always presented in the third position. Thus, the decrement of mean RT indicates the existence of serial order learning, rather than familiarization with a single symbol. In the BR condition, target detection in the 100% and blank conditions was faster than in the novel condition. Target detection in the 50% condition was not facilitated. These results suggest that the facilitation of serial order learning was disrupted when the symbol sequence was not fixed, as in Experiments 1 and 2. In the NBR condition, however, faster target detection was found in all three conditions (100%, 50%, and blank). This result matches with that of Experiment 2, where the interference by the subsequent symbol did not occur when the symbols were presented supraliminally.

## EXPERIMENT 4

In the BR condition, interference from the subsequent symbol was attenuated only when the transition probability was 100%. The sequence with the transitional probability of 50% could not be learned when the participants could not detect the stimuli. Meanwhile, only the transition probability between the target and the subsequent symbols was manipulated in Experiment 3. To verify the hypothesis that learning of transition probability occurs only when the participants can detect the stimuli, we need to examine the effect of transition probability on the target and prior symbols.

**FIGURE 6:**
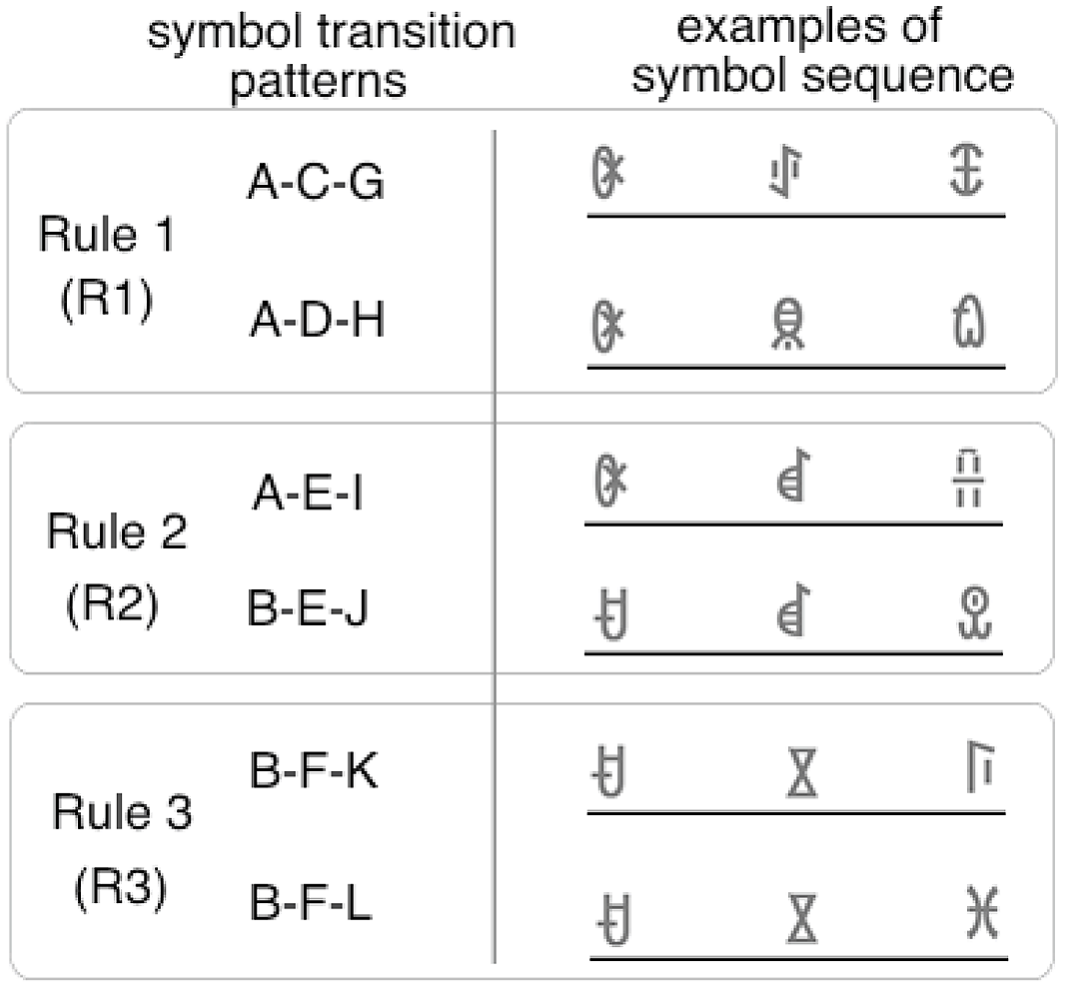
**Examples of the symbol sequence in the familiarization phase of Experiment 4.** Eighteen types of triplets, including the examples above, were used in the familiarization phase. Target symbols appeared only in the third triplet position both in familiarization and test phases. Each triplet was constructed according to one of three rules. In the rule 1 (R1) condition, the target symbol can be predicted by the second symbol (if C appears, G always follows). In the rule 2 (R2) condition, the target symbol can be predicted by the combination of the first and the second symbols (if B and E appear in succession, J always follows). In the rule 3 (R3) condition, the target symbol cannot be predicted deterministically (if B and F appear in succession, K or L follows).

In this experiment, the symbol sequence consisted of triplets. The target position was fixed to the third position. The first and second symbols were chosen according to one of the three transition rules (**Figure 6**). The rules were set to manipulate the transition probability between the target and prior symbols. In the rule 1 (R1) condition, the transition probability between the second and third symbol was set to 100% (e.g., A-C-G or A-D-H). Thus, the third symbol could be predicted perfectly by the second symbol. In the rule 2 (R2) condition, the transition probability between the prior two symbols and the third symbol was set to 100%. Thus, the third symbol could be predicted perfectly by the combination of the first and second symbols (e.g., A-E-I or B-E-J). In the rule 3 (R3) condition, the transition probability between the prior two symbols and the third symbol was set to 50% (e.g., B-F-K or B-F-L). Thus, the third symbol could not be predicted deterministically.

If serial order learning under BR occurs only between adjacent symbols, facilitation in target detection will be found only in the R1 condition. If the learning mechanism under BR can utilize information about the combination of the first and second symbol, facilitation in the R2 condition will also be observed. If the transition probability between the prior and target symbols can be learned, facilitation in the R3 condition will be found because, in this condition, the chance of predicting the target is 50%.

### METHODS

#### Participants

Fifty-six undergraduate students participated (aged 19–22, average 19.2 years); 24 students participated in the NBR group (10 males), and the other 32 participated in the BR group (16 males). All were native Japanese speakers and had normal or corrected-to-normal vision. None had participated in Experiments 1, 2, or 3.

#### Stimuli and procedure

The stimuli and procedures were the same as those in Experiment 1, with the following exceptions: (1) Target symbols appeared only in the triplets’ third position in both the familiarization and the test phases. In the test phase, the novel symbol’s position was fixed at the third position. (2) Each triplet was constructed according to one of the three rules (R1, R2, or R3), and 18 types of Yi script triplets were used for the familiarization phase. The stimuli consisted of 42 different symbols (36 symbols for familiarization and 6 novel symbols).

### RESULTS

**FIGURE 7:**
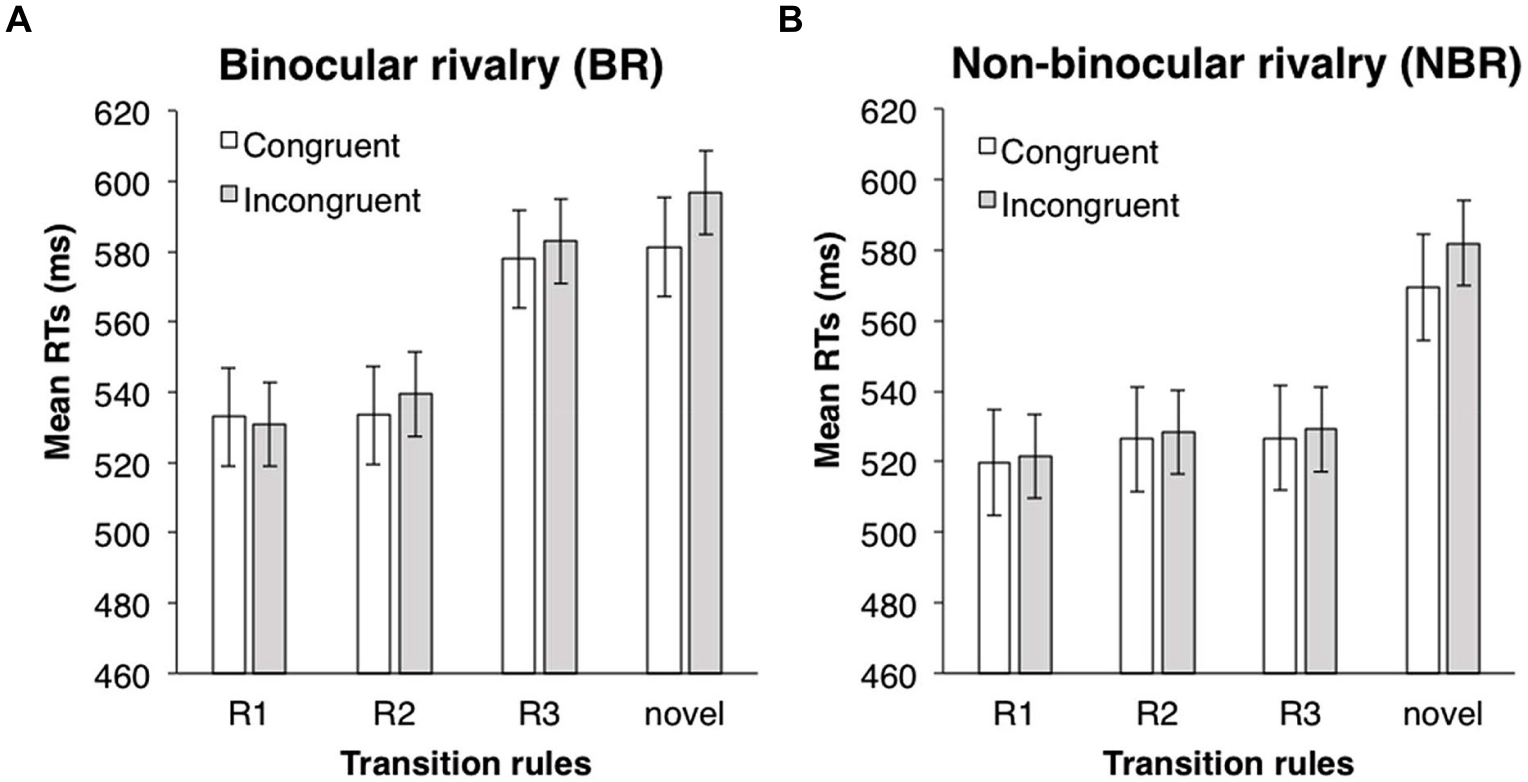
**Mean RTs of target detection in Experiment 4. (A)** BR condition. **(B)** NBR condition. White bars indicate the mean RTs in the congruent eye condition; gray bars indicate the mean RTs in the incongruent eye condition. Error bars represent SE.

ANOVAs (one between-subject and two within-subject design) were performed with a 2 (suppression: BR vs. NBR) × 2 (eye congruency: congruent vs. incongruent) × 3 (transition rules: R1, R2, and R3) design. Incorrect responses and responses with an RT below 200 ms (3.0%) were excluded from the analysis. In each trial, the RT was converted by a logarithmic transformation, because the distribution of all data was positively skewed (1.80). Mean RTs for each condition are shown in **Figure 7**. The main effect of transition rules was significant [*F*(2,108) = 6.59, *p* < 0.001, partial η^2^ = 0.11], and the main effect of suppression was marginally significant [*F*(1,54) = 3.34, *p* < 0.1, partial η^2^ = 0.06]; however, the main effect of eye congruency was not [*F*(1,54) = 0.08, *n.s.*]. The interaction between suppression and transition rules was significant [*F*(2,108) = 4.10, *p* < 0.05, partial η^2^ = 0.07]. ANOVAs for simple main effects revealed that the main effect of transition rules was significant in the BR condition, but not in the NBR condition [BR condition: *F*(2,62) = 14.12, *p* < 0.001, partial η^2^ = 0.31; NBR condition: *F*(2,46) = 0.17, *n.s.*]. Multiple comparison (Shaffer’s modified sequentially rejective Bonferroni procedure, *p* < 0.05) was conducted on the main effect of transition probability in the BR condition. There was a significant difference between the R1 and R3 conditions [R3 > R1, *t*(31) = 5.40, *d* = 0.60] and between the R2 and R3 conditions [R3 > R2, *t*(31) = 3.36, *d* = 0.55]. In the R3 condition, the mean RT was significantly longer than in other transition probability conditions. However, in the NBR condition, transition probability had no significant effect. The tendency that RTs were shorter when the presentation eyes were congruent was only marginally significant none of the other interactions were significant [suppression × eye congruency: *F*(1,54) = 0.01, *n.s.*; eye congruency × transition rules: *F*(2,108) = 0.05, *n.s.*; three-way: *F*(2,108) = 0.003, *n.s.*].

To confirm the learning effect, RTs in the R1, R2, and R3 conditions were compared with RTs for the novel symbols via paired two-tailed *t*-tests (eye congruency conditions were collapsed). In the BR condition, RTs in R1 and R2 conditions were significantly shorter than in the novel symbols; RTs in the R3 condition did not differ from those of the novel symbols [R1 vs. novel: *t*(62) = 3.03, *p* < 0.05, *d* = 0.76; R2 vs. novel: *t*(62) = 2.81, *p* < 0.05, *d* = 0.70; R3 vs. novel: *t*(62) = 0.01, *n.s.*]. In the NBR condition, RTs in all three conditions were significantly shorter than in the novel symbols [R1 vs. novel: *t*(46) = 2.60, *p* < 0.05, *d* = 0.75; R2 vs. novel: *t*(46) = 2.09, *p* < 0.05, *d* = 0.60; R3 vs. novel; *t*(46) = 2.57, *p* < 0.05, *d* = 0.74]. The effect of learning was observed except in the R3 condition under BR.

ANOVAs were performed on the angular transformed percentage of incorrect responses for each condition. Neither main effects nor interaction was significant [BR; *F*(1,54) = 0.15, *n.s.*, eye congruency; *F*(1,54) = 0.32, *n.s.*; transition rules; *F*(2,108) = 0.96, *n.s.*; suppression × eye congruency: *F*(1,54) = 0.32, *n.s.*; suppression × transition rules: *F*(2,108) = 0.18, *n.s.*; eye congruency × transition rules: *F*(2,108) = 2.00, *n.s.*; three-way: *F*(2,108) = 0.01, *n.s.*]. There was no evidence of speed–accuracy trade-off or effects of task difficulty on RT.

We counted the number of correct responses in the perceptibility test. The average percentage of correct responses in the BR condition was 53%, not significantly higher than chance level [*t*(31) = 1.24, *n.s.*].

### DISCUSSION

In the BR condition, target detection was facilitated only in the R1 and R2 conditions. Target detection in the R3 condition was slower than in the R1 and R2 conditions; it was not faster for the novel symbols. This suggests that facilitation under BR does not occur when the target symbol cannot be predicted deterministically. In the NBR condition, however, target detection in all three conditions (R1, R2, and R3) was facilitated. These results confirmed the hypothesis that learning on the transition probability occurs only when the participants can detect the stimuli. Under BR, facilitation was observed not only in the R1 condition but also in the R2 condition. The suggestion is that the learning mechanism under BR can utilize information about the combination of the prior two symbols.

## GENERAL DISCUSSION

### SERIAL ORDER LEARNING UNDER BR

We investigated serial order learning under BR and found consistent evidence of serial order learning of subliminal stimuli. The detection latency for the second symbol (Experiment 1) or the second and third symbols (Experiment 2) was faster than that for the first symbol. As far as we know, this is the first evidence of serial order learning under a condition wherein participants cannot detect the stimuli.

We also found that detection latency for the last symbol was delayed (Experiments 1 and 2). The fact that this phenomenon was observed in Experiment 1, using triplets, and Experiment 2, using quadruplets, confirmed that the delay was not due to a limitation of the learning mechanism’s memory span. The phenomenon was observed only when the perception of stimuli was suppressed (BR condition). The fact that the phenomenon was not observed in the NBR condition, where the test phase procedure was the same as that of the BR condition, suggests that the delay was caused by interference in the familiarization phase under BR. Serial order learning in the familiarization phase seemed to be disturbed by interference under BR.

In Experiments 3 and 4, we examined the effect of the symbol sequences’ transition probability. In Experiment 3, target detection was not facilitated when the transition probability of the next symbol was not fixed (50% condition). This result supports the view that learning is disturbed by interference caused by the subsequent symbol. However, in the NBR condition, learning occurred even when the symbol sequences were not fixed. In Experiment 4, we examined the effect of the transition probability of the prior and target symbols. Target detection was facilitated only when the target symbol could be predicted deterministically (R1 and R2 conditions) under BR. In the NBR condition, however, learning occurred even when the target symbol could not be deterministically predicted according to the prior symbols. These results indicate that learning on symbols’ transition probability occurs only when participants can see stimuli.

### MECHANISM OF THE SUBLIMINAL SERIAL ORDER LEARNING AND INTERFERENCE

In the test phase, no flash patterns were presented in either BR or NBR conditions. The participants could thus detect the target in the test phase, where each symbol was presented for 400 ms. However, the detection delay of the last symbol was found only in the BR condition. The suggestion is that the delay occurred during the familiarization phase. It is inferred that learning in the familiarization phase is disrupted by the spatiotemporal variation of the symbols when the symbol sequence is not fixed.

Orientation detectors in V1 possess direction sensitivity; i.e., some complex cells respond only when the stimulus (bar) moves in one direction and not when it moves in the opposite direction (Hubel and Wiesel, 1962). These detectors are considered to be tuned to spatiotemporal patterns (Adelson and Bergen, 1985). When a visual symbol is replaced by another symbol in the same screen position, these detectors will respond because spatiotemporal change emerges as a result of the symbols’ replacement. In addition, these detectors change their tuning to increase their discriminatory power to the current input patterns (Clifford and Ibbotson, 2002).

Serial order learning of a fixed sequence under BR might depend on adaptation of the spatiotemporal tuning of these detectors. Notably, all symbol images used in our experiments were blurred to prevent conscious perception in the BR condition. The interference by the subsequent symbol might have a strong effect because of the degraded stimuli and zero ISI between the symbols. We presume that familiarization with the fixed symbol sequence reduces the interference by the latter symbol because the spatiotemporal tuning of feature detectors are adapted to the symbols’ transition. These detectors can be considered the “transition detector” of the two consecutive symbols.

When symbol A replaces symbol B on the screen, a specific spatiotemporal pattern emerges. The transition detector adapts to this specific transition pattern. If a participant is being familiarized with the two symbol sequences of A-B-C-D and A-B-C-E, when A is presented on the screen, the A-B transition detector is activated. This facilitates the perception of symbol B. When B is presented, the A-B transition detector remains active and activates the B-C transition detector. When C is presented, the B-C transition detector remains active, and both C-D and C-E transition detectors become active. The two activated detectors (C-D and C-E) compete with each other because they share the same retinal position and temporal position. As a result, the perception of C is disturbed. We conjecture that this competition and inhibition process is the source of the backward interference found in our experiments. On the contrary, the perception of B is not disturbed because the B-C transition detector does not compete with other detectors. The process above explains our results in the BR condition. In Experiments 1 and 2, the target detection for the second symbol (B in the example above) was facilitated. The target detection in the 50% condition of Experiment 3 (C in the example above) was not facilitated. Note that we did not directly observe the effect of the interference in the familiarization phase; instead, we inferred the existence of the interference from the detection latency delay of the last symbol during the test phase. To understand the mechanism of interference and learning, future research should examine the nature of the perceptual masking effect under BR.

The findings of Experiment 4 suggests that transition detectors can adapt to a longer sequence. After the participants were familiarized with triplets such as A-E-I and B-E-J under BR, the detection of the target I or J was facilitated in the test phase (R2 condition in Experiment 4). This type of facilitation cannot be explained by adaptation to the two adjacent symbols and suggests that the transition detectors have a longer temporal receptive field. When the transition to the third (target) symbol was not fixed (R3 condition in Experiment 4), target detection was not facilitated. These results suggest that the transition across three symbols can be learned under BR if the transition to the target is deterministic. However, whether learning occurs on all three symbols or the combination of the first and the third symbol remains unknown.

One might notice that triplets used in the R2 condition of Experiment 4 (A-E-I and B-E-J) resemble those used in the 50% condition of Experiment 3 (A-B-C-D and A-B-C-E). If the target had been the second triplet symbol in Experiment 4, target detection in the R2 condition would not be facilitated because subsequent symbols were varied. It is intriguing that the detection of varying symbols (I or J in the example above) was facilitated. These results indicate that interference acts only backward. The competition between the transition detectors (E-I and E-J) perhaps does not disturb the perception of the latter symbol.

Interference from subsequent symbols was not observed in the NBR condition. This result was similar to that of Kim et al. (2009), where stimuli were presented supraliminally and statistical learning of RSVP sequence was observed. The process of statistical learning can be explained by a recurrent neural network model (Elman, 1990). The recurrent network acquires a representation of the input pattern sequence, through learning, to predict the next input. When the network learns to predict the next symbol in the sequence, multiple units (artificial neurons) are simultaneously activated in the intermediate (hidden) layer. The activation of each unit in the layer can be regarded as an individual hypothesis about the next input. The recurrent network can replicate the monotonic decrease of detection latency by statistical learning (Elman, 1990). The learning of transition probability, which was observed when the participants could detect the stimuli in our experiments, possibly depends on a learning mechanism similar to the recurrent network involved in higher order cortical function. It is conceivable that each symbol transition is represented as an independent hypothesis at the higher level processing. If symbols are represented at the abstract level where their physical properties are discarded, representations of various types of symbol transitions can be simultaneously activated even when they conflict with each other at the physical level, as in the recurrent neural network. Such representation enables statistical learning of the sequences’ transition probability. In visual areas, where spatiotemporal processing of visual stimuli is conducted, symbol transitions might be represented as competing hypotheses because the representation depends on the particular spatial and temporal position. This type of representation is not suitable for statistical learning because it cannot hold various hypotheses on the same input. We argue that under BR, this is the cause of statistical learning’s absence on the transition probability of sequences.

### MULTISTAGE LEARNING OF THE SERIAL ORDER OF VISUAL STIMULI

The results of our experiments suggest that multiple processes are involved in learning during the familiarization phase. We propose that at least three stages of learning processes are involved in serial order learning (**Table 2**).

**Table 2:** Three types of learning process suggested by our experiments.

Subliminal		Supraliminal
Monocular	Binocular	
Adaptation to global visual properties	Learning on fixed transition of the symbols	Learning on probabilistic transition of the symbols

The first stage, which lies at the monocular level of early visual processing, consists of feature detectors that respond to static visual features. These detectors adjust their tuning to global visual properties, such as spatial frequency, and respond quickly to the stimuli that share those properties. The facilitation effect depends on eye congruency between familiarization and test phases because this stage lies at the monocular level. The experimental results suggest that the effect of first stage learning acts additively to that of other stages.

The second stage lies at the binocular level of visual processing. It consists of “transition detectors” that respond to the spatiotemporal input pattern. These detectors adapt their tuning to the symbols’ fixed transition. As previously discussed, both serial order learning and detection delay of the last item are explained by the processing in this stage.

The third stage involves the input from both eyes and is a higher order level of perceptual processing. Although the first and second stages occur under BR, the third stage, in which the symbol sequence’s transition probability is learned, does not. This suggests that stimuli must be consciously attended to in order to learn the transition probability (Turk-Browne et al., 2005; Kim et al., 2009). Future research is required to examine the functional role of visual attention.

The second stage’s contribution to serial order learning is our study’s novel finding. By investigating the learning process under BR, we could differentiate the second stage from the other stages. It would not be feasible to suggest that the second stage works only under BR. Probably, the second stage is a process of conscious learning, but we do not notice it because it acts additively on the third stage.

## CONCLUSION

We investigated whether serial order learning occurred when participants could not consciously detect stimuli. Triplets of novel symbols were presented in succession in the familiarization phase of Experiment 1. The perception of symbols was suppressed by CFS, which establishes stable BR (Tsuchiya and Koch, 2005; Tsuchiya et al., 2006). In the test phase, the detection latency for the second symbol was faster than that for the first symbol. This result indicates that serial order learning occurs even when participants cannot detect the stimuli. We also found that overall target detection latency was shortened when symbols were presented to the same eye in both the familiarization and test phases. This suggests that adaptation to global visual properties occurs at monocular level processing, which lies in the early visual area. Quadruplets of symbols were used in Experiment 2. The detection latency for the first, second, and third symbols decreased with the serial position; this confirmed that serial order learning occurred under BR. The detection of targets located in the last position of the triplets (Experiment 1) or quadruplets (Experiment 2) was slowed, suggesting that target detection was disturbed when subsequent symbols were not fixed. Thus, the effect of the symbol sequence’s transition probability was examined in Experiments 3 and 4, which showed that serial order learning under BR occurs only when the symbol transition is fixed. When the participants could detect the symbol sequence in the familiarization phase, the transitional probability of the varying symbol sequence could be learned. These results suggest that serial order learning under BR takes place at a relatively lower level of the brain and that learning of transitional probability is conducted in a higher level. We argue that at least three types of learning processes (monocular, binocular/subliminal, and binocular/supraliminal) underlie serial order learning of novel visual symbols. We infer that the binocular/subliminal learning process depends on the adaptation of feature detectors that respond to spatiotemporal patterns, which lie in the visual cortex. By directly manipulating the stimuli’s spatiotemporal properties, future researchers should verify this hypothesis.

## Conflict of Interest Statement

The authors declare that the research was conducted in the absence of any commercial or financial relationships that could be construed as a potential conflict of interest.
